# Editorial: Neutrophil function and dysfunction: pathways, impact, and therapeutic insights

**DOI:** 10.3389/fimmu.2026.1887933

**Published:** 2026-06-15

**Authors:** Baochen Fang, Mengjie Li, Yiwei Zhu, Alan Y. Hsu, Lihui Guo, Zhifeng Chen, Minhao Liu, Hui Yu, Hongli Xu, Zhicheng Peng, Hui Chen

**Affiliations:** 1 Department of Plant Sciences, North Dakota State University, Fargo, ND, United States; 2 Department of Chemistry and Biochemistry, Texas Tech University, Lubbock, TX, United States; 3 Vatche and Tamar Manoukian Division of Digestive Diseases, Department of Medicine, David Geffen School of Medicine, University of California, Los Angeles, Los Angeles, CA, United States; 4 Department of Microbiology and Immunology, Yong Loo Lin School of Medicine, National University of Singapore, Singapore, Singapore; 5 NUS Yong Loo Lin School of Medicine (NUSMED) Immunology Translational Research Programme, Yong Loo Lin School of Medicine, National University of Singapore, Singapore, Singapore; 6 Immunology Programme, Life Science Institute, National University of Singapore, Singapore, Singapore; 7 Ragon Institute of Mass General Brigham, MIT, and Harvard University, Boston, MA, United States; 8 Department of Experimental Immunology, Amsterdam Infection and Immunity Center, Amsterdam University Medical Centers (UMC), Location Academic Medical Center (AMC), University of Amsterdam, Amsterdam, Netherlands; 9 Institute for Biological Therapy, Henan Academy of Innovations in Medical Science, Zhengzhou, Henan, China; 10 Department of Data Science & AI (DSAI), Monash University, Clayton, VIC, Australia; 11 Institute of Research and Clinical Innovations, Neusoft Medical Systems Co., Ltd., Hangzhou, Zhejiang, China; 12 Department of Cancer Biology, Abramson Family Cancer Research Institute, Perelman School of Medicine at the University of Pennsylvania, Philadelphia, PA, United States; 13 The Department of Hematology, Affiliated Hospital of Nanjing University of Chinese Medicine, Nanjing, Jiangsu, China; 14 Hubei Cancer Hospital, Tongji Medical College, Huazhong University of Science and Technology, Wuhan, Hubei, China; 15 Department of Pathology, Harvard Medical School, Department of Lab Medicine, Children’s Hospital Boston, and Dana-Farber/Harvard Cancer Center, Boston, MA, United States

**Keywords:** granule, immune dysfunction, inflammatory death, innate immunity, neutrophils, therapeutic targeting, tissue injury

Neutrophils are terminally differentiated and the most abundant circulating leukocytes of the innate immune system. They are rapidly recruited to sites of infection or tissue injury, where they contribute to host defense through phagocytosis, degranulation, and the release of neutrophil extracellular traps (NETs) (1). These rapid-response cascades are supported by a highly specialized intracellular granule system, including primary (azurophilic), secondary (specific), tertiary (gelatinase), and secretory granules (2). These subcellular compartments enable the tightly regulated mobilization of antimicrobial enzymes, proteases, and inflammatory mediators that are essential for the clearance of pathogenic microorganisms and immune regulation. However, once neutrophils are excessively activated or dysregulated, granule integrity is impaired, lysosomal membrane permeabilization (LMP) is induced, and the granular proteins are continuously released into the cytoplasm and even further into the extracellular matrix, ultimately driving pro-/non-inflammatory death and tissue injury (3). Dysregulated neutrophil responses have been implicated in a broad range of diseases, including chronic inflammatory disorders, oncology, autoimmune diseases, systemic inflammatory syndromes, acute tissue injury, and fibrosis (4, 5). Generally, it is considered that degranulation plays the primary role in regulating granule dynamics, antimicrobial defense, and collateral tissue damage. Recently, Peng et al. identified an autophagy-dependent granule degradation and vacuolation in over-activated neutrophils due to the elevated palmitic acid levels (6). Except for degranulation, the study provides new insight into how autophagy regulates granule homeostasis and neutrophil function. Our new data and previous studies demonstrate that aberrant degranulation, altered granule homeostasis, and dysregulated neutrophil spontaneous death contribute to uncontrolled systemic inflammation, tissue damage, and fibrosis (1, 5, 7, 8). Accordingly, precise regulation of neutrophil activation and fate is critical not only for antimicrobial defense but also for limiting collateral tissue injury and maintaining immune homeostasis.

Neutrophils respond dynamically to diverse environmental cues associated with various diseases and exhibit significant phenotypic heterogeneity (9, 10). Ng et al. investigated that neutrophils display substantial phenotypic, functional, and developmental heterogeneity in both health and disease, and highlighted the potential mechanisms that shape neutrophil diversity, including regulatory and genomic programs (10). Neutrophil heterogeneity can be exploited in various diseases, such as cancer, to promote pathology. Therefore, Neutrophil profiling may have diagnostic and prognostic value across multiple disease contexts (11–14). To fulfill their dual role in immunity and tissue injury, neutrophils are programmed to be short-lived effector cells, ideally maintaining a balance between robust host defense and systemic tissue damage. Neutrophils undergo several regulated cell death programs, including apoptosis, pyroptosis, necrosis, NETosis, and other lytic pathways, each contributing differently to inflammation resolution or amplification (8, 15–17). Consequently, an increasing number of studies are focusing on modulating the balance between these fates to investigate how neutrophils promote the resolution or perpetuation of inflammation. For example, Hsu et al. identified a novel CD55-positive large aging neutrophil-derived vesicles (LAND-Vs), which suppress complement activation, reduce neutrophil recruitment, and limit tissue damage, thereby promoting inflammation resolution (18). This finding indicates neutrophilic inflammation, or the tissue damage signal, cannot be allowed to escalate indefinitely. Increasingly, neutrophils are recognized as active regulators of tumor microenvironment remodeling, fibrosis, and tumorigenesis (1, 8, 12). Therefore, neutrophils are currently being developed as an adjunctive cancer therapy and diagnostic tool. In cancer, neutrophils either support or suppress tumor progression by shaping the tumor microenvironment and influencing anti-tumor immunity (19, 20). The characteristic neutrophil-mediated tissue damage is being strategically harnessed to promote the infiltration of CAR T cells into solid tumors. In diagnostics, emerging artificial intelligence (AI) technologies, particularly AI-enabled point-of-care testing (POCT), are further expanding the application of neutrophil heterogeneity (21). Together, these advances are positioning neutrophils as central players across diverse physiological and pathological processes, with growing relevance for various disease diagnostics, neutrophil-mediated tissue/organic damage, fibrosis, anti-inflammation, and anti-tumor therapeutic development.

Neutrophils possess distinctive structural and functional characteristics that continue to reveal new biology. Despite major advances in understanding neutrophils, research into neutrophil function is continually expanding into new areas, and many previous findings are being revised in light of discoveries. For instance, reactive oxygen species (ROS) are crucial for NET formation. Björnsdottir et al. demonstrated that only blocking intragranular MPO activity or MPO-processed ROS effectively prevents PMA-induced NETosis (22). The study provides new insight that intracellular granule-localized ROS signaling is essential for NET formation. Intriguingly, Silvestre-Roig et al. discovered that the spleen can perform rapid stress-induced granulopoiesis to produce fully functional, infection-fighting neutrophils in a type I interferon-dependent manner, revealing a critical extramedullary mechanism that safeguards host defense during persistent inflammation (23). Importantly, Vicanolo et al. identified a specialized population of matrix-producing neutrophils in the skin that reinforces barrier integrity and forms TGFβ-dependent extracellular matrix “rings” around wounds to physically prevent bacterial invasion (24). These findings have substantially advanced the field. However, the exploration of neutrophils is far from over. Many fundamental mechanisms remain unresolved, particularly how neutrophils are regulated in disease-specific contexts and how their functions can be selectively targeted without compromising host defense. In this context, we aim to synthesize emerging findings on neutrophil biology here to guide future research and therapeutic development. This Research Topic aims to provide an integrated and mechanistic understanding of neutrophil biology in health and disease settings, with particular emphasis on the crucial role of neutrophil heterogeneity in diagnosis and prognosis, and neutrophil dysregulation shapes diverse pathophysiological outcomes. By bringing together mechanistic, multi-omics, and translational studies across conditions such as trauma, sepsis, COVID-19, autoimmune and inflammatory disorders, fibrosis, reproductive diseases, and impaired wound healing, the Research Topic seeks to define the key molecular and cellular pathways governing neutrophil activation, functional reprogramming, and cell fate decisions. Ultimately, the long-term aims of this Research Topic are to reveal the distinct neutrophil states during various disease progression and resolution, to enable precise modulation of neutrophil-driven pathophysiology.

Neutrophils have long been viewed as short-lived first responders, mediators of inflammation, and primary culprits behind tissue damage. However, the underlying mechanisms still await unraveling. The studies collected in this Research Topic collectively reinforce a clearer picture: neutrophils are highly plastic, context-dependent regulators of inflammation whose functional states shape outcomes across infectious, autoimmune, and chronic inflammatory diseases. In acute inflammatory settings, including trauma and sepsis, neutrophil heterogeneity emerges as a key determinant of disease severity. Vlkova et al. describe distinct neutrophil subpopulations after trauma, where immature and CD16^low^CD62L^low^ cells are associated with systemic inflammation and metabolic stress, while hypersegmented neutrophils appear more linked to resolution and recovery. In parallel, Langston et al. extend this concept to sepsis, using proteomic network modeling to define hyperimmune, hypoimmune, and hybrid neutrophil phenotypes, each with distinct druggable pathways and potential for phenotype-guided therapeutic repurposing. A similar idea of transcriptional and functional diversity is reinforced in infectious inflammation. Ito et al. show that in COVID-19-associated acute respiratory distress syndrome (ARDS), neutrophil gene expression programs stratify patients with markedly different clinical trajectories, particularly in relation to ventilator dependency, suggesting that neutrophil transcriptomic states may serve as prognostic indicators rather than simple markers of inflammation.

In autoimmune and immune-mediated diseases, neutrophils emerge as active contributors to tissue injury and dysregulated immunity. Riaz et al. demonstrate that partial loss of endoplasmic reticulum aminopeptidase 1 (ERAP1) aggravates DSS-induced colitis and alters treatment responses to sulfasalazine, highlighting how antigen processing pathways modulate neutrophil-associated inflammation in ulcerative colitis. Zhao et al. further show that blood heparin-binding protein reflects systemic neutrophil activation across autoimmune kidney diseases, linking neutrophil activity to disease severity in an estimated glomerular filtration rate (eGFR)-independent manner. In reproductive immunology, He et al. identify a TNF-α-polarized neutrophil state and amyloid precursor protein (APP)-CD74 signaling axis in recurrent miscarriage, with Chemokine (C-X-C motif) ligand 1 (CXCL1) emerging as a potential diagnostic and mechanistic biomarker of immune-mediated trophoblast injury.

Beyond classical inflammation, neutrophils also play central roles in chronic tissue remodeling and repair failure. Liang et al. summarize how neutrophil infiltration, elastase release, and NET formation drive pulmonary fibrosis through sustained crosstalk with epithelial cells, macrophages, and fibroblasts, reinforcing fibrosis as an immunologically active process rather than a purely structural disease. In wound healing, Deng et al. provide direct experimental evidence that excessive NET formation impairs postoperative anal fistula repair, while pharmacological inhibition of the Nox4/ROS/PI3K/Akt/PADI4 axis restores healing and reduces bacterial burden without compromising host defense. Mechanistic insights into NET regulation are further expanded by Oliveira et al., who describe a self-amplifying feedback loop in which S100 calcium-binding proteins and histones propagate NET formation through the receptor for advanced glycation end products (RAGE) and toll-like receptor 2 (TLR2) signaling, while albumin acts as a physiological buffer that restrains excessive NETosis. This framework places NET formation within a tightly controlled network of endogenous activators and inhibitors, rather than a simple binary antimicrobial response.

Taken together, these studies reshape the view of neutrophils from uniform effector cells into highly dynamic and context-dependent regulators of immunity and tissue injury. Across infection, autoimmunity, trauma, reproductive failure, and fibrotic disease, neutrophil states consistently correlate with clinical outcomes and, in several cases, define actionable therapeutic or diagnostic targets. Importantly, multiple studies in this Research Topic also point toward a shift from broad immunosuppression toward more refined, phenotype- or pathway-specific modulation of neutrophil activity. Overall, this body of work highlights a clear direction for the field: understanding neutrophil dysfunction is no longer sufficient at the level of cell counts or generic activation markers. Instead, integrating functional phenotyping, transcriptomics, proteomics, and signaling network analysis is beginning to reveal clinically meaningful neutrophil states that can be leveraged for diagnosis, prognosis, and therapy. Within this evolving framework, neutrophils stand at the center of a unifying immunological axis linking acute inflammation, chronic disease progression, and tissue repair, offering both challenges and opportunities for future translational intervention.
